# Increased ROS-Dependent Fission of Mitochondria Causes Abnormal Morphology of the Cell Powerhouses in a Murine Model of Amyotrophic Lateral Sclerosis

**DOI:** 10.1155/2021/6924251

**Published:** 2021-10-14

**Authors:** Jan Stein, Bernd Walkenfort, Hilal Cihankaya, Mike Hasenberg, Verian Bader, Konstanze F. Winklhofer, Pascal Röderer, Johann Matschke, Carsten Theiss, Veronika Matschke

**Affiliations:** ^1^Department of Cytology, Institute of Anatomy, Medical Faculty, Ruhr University Bochum, D-44801 Bochum, Germany; ^2^Electron Microscopy Unit, Imaging Center Essen, Medical Faculty of the University of Duisburg-Essen, D-45147 Essen, Germany; ^3^Department of Molecular Cell Biology, Institute of Biochemistry and Pathobiochemistry, Medical Faculty, Ruhr University Bochum, D-44801 Bochum, Germany; ^4^Institute of Cell Biology (Cancer Research), University Hospital Essen, University of Duisburg-Essen, D-45147 Essen, Germany

## Abstract

Amyotrophic lateral sclerosis (ALS) is the most common motor neuron disease in humans and remains to have a fatal prognosis. Recent studies in animal models and human ALS patients indicate that increased reactive oxygen species (ROS) play an important role in the pathogenesis. Considering previous studies revealing the influence of ROS on mitochondrial physiology, our attention was focused on mitochondria in the murine ALS model, wobbler mouse. The aim of this study was to investigate morphological differences between wild-type and wobbler mitochondria with aid of superresolution structured illumination fluorescence microscopy, TEM, and TEM tomography. To get an insight into mitochondrial dynamics, expression studies of corresponding proteins were performed. Here, we found significantly smaller and degenerated mitochondria in wobbler motor neurons at a stable stage of the disease. Our data suggest a ROS-regulated, Ox-CaMKII-dependent Drp1 activation leading to disrupted fission-fusion balance, resulting in fragmented mitochondria. These changes are associated with numerous impairments, resulting in an overall self-reinforcing decline of motor neurons. In summary, our study provides common pathomechanisms with other ALS models and human ALS cases confirming mitochondria and related dysfunctions as a therapeutic target for the treatment of ALS.

## 1. Introduction

Amyotrophic lateral sclerosis (ALS) is the most common systemic disease of the motor system. The annual incidence rate in Europe is 2.2 per 100,000 inhabitants per year [1]. It is mainly classified in a familial and sporadic form. The sporadic form of ALS (sALS) is clearly the most frequent with a share of about 90-95%, followed by the familial form (fALS) with about 5-10%. The disease is characterized by bilateral degeneration of cells from *tractus corticospinalis*, in the nuclei of cranial motoric nerves as well as motor neuronal cells in the anterior horn of the spinal cord [2]. The degeneration of the first motor neuron leads to progressive spastic paresis and painful muscle cramps combined with hyperreflexia, pseudobulbar paralysis, and pyramidal path signs [3]. In the course of time, these symptoms are masked by the degeneration of the second motor neuron. The main symptoms are muscular atrophy and weakness, fasciculation (especially of the tongue), and progressive respiratory insufficiency [4]. Ultimately, respiratory failure leads to death within 2 to 5 years after diagnosis in most cases. Although the disease was already described and diagnosed in 1869, no curative therapy has been developed to date [5]. Supportive medications like Riluzole, which has antiglutamatergic effects, and Edavarone, which is supposed to reduce oxidative stress, can only prolong survival by a few months [6].

Only a few genes have been associated with ALS as causative factors so far. The most frequently altered genes in ALS patients are *C9orf72*, *SOD1*, *TARDBP*, and *FUS* [7, 8]. And new mutations are constantly being linked to ALS, such as mutations in profilin 1 (*PFN1*) [9], specific kinesin isoforms (*KIF5A*) [10], or the chaperon Sigma-1 receptor (*SIGMAR1*) [11]. However, the genetic origin of most ALS cases remains unclear [7]. In the sporadic form, even more than 80% of ALS cases remain without an uncovered genetic cause. However, current knowledge suggests that ALS is caused by a complex interplay of different pathomechanisms, including the motor neurons themselves and interactions with neighboring cells such as microglia and astrocytes [12]. At a cellular level, protein misfolding and aggregation, glutamate-induced excitotoxicity, neuroinflammation, and deficient axonal transport are among the known pathological events in ALS [13]. In recent years, mitochondrial dysfunction and oxidative stress have increasingly become the focus of science attention and are now considered to play a key role in the pathogenesis cascade of ALS [14].

In several models used to study ALS pathology, proteins of the antioxidative system and oxidative stress play important roles. An example for this is the murine ALS model of the superoxide dismutase 1 (SOD1) [15]. About 2.5-23% of fALS and 0.44-7% of sALS cases are related to this *SOD1* gene, which encodes for a cytosolic antioxidative enzyme [16]. Further studies in tissues from ALS patients and animal models have demonstrated dysfunctional mitochondria with consequently increased oxidative stress, as reviewed in Carrì et al.'s study [17]. This is not only present on functional but also on morphological level. Based on *postmortem* studies from ALS patients and transgenic SOD1^G93A^ mice, fragmentation of the mitochondrial network is described on a morphological level [18, 19]. Deregulated fission and fusion-related enzymes like mitochondrial Dynamin-like 120 kDa protein (Opa1), Mitofusin1 (Mfn1), mitochondrial fission 1 protein (Fis1), and Dynamin-1-like protein (Drp1) were detected as reasons for the fragmentation in SOD1^G93A^ transgenic mice [20]. This underlines the importance of the fusion/fission balance and oxidative stress in the pathogenesis of ALS.

For our investigations, we used the wobbler mouse as an ALS model. The wobbler mouse was first described by Falconer in 1956 and arose from the C57BL/Fa mouse strain by a spontaneous mutation. Later, a loss-of-function mutation in the *VPS54* gene was identified as the genetic cause of the wobbler ALS disease [21]. Recently, several mutations of *VPS54* have been discovered in human ALS patients with Project MinE (http://databrowser.projectmine.com/, Accessed 11 May 2021) indicating an involvement of this gene in the pathogenesis of some ALS cases. Nearly all phenotypic and cellular symptoms of ALS, like motor defects, tremor, muscle weakness and atrophy, degeneration of the 1st and 2nd motor neuron, astrogliosis, defects in vesicle transfer, and axonal transport, are present in homozygous wobbler mice [22–24]. Wobbler disease develops within three typical stages—presymptomatic (p0-p19), evolutionary (p20-p39), and stable (>p40) stage. In the presymptomatic stage, both genotypes show no clinically visible differences. In the evolutionary stage, typical symptoms such as head tremor, motor defects, and muscle weakness develop rapidly. At a cellular level, degeneration of the upper and lower motor neurons, reduced axonal transport, and mitochondrial dysfunction can be observed [24, 25]. In the stable stage starting from p40, the symptoms stagnate [26]. Mitochondrial dysfunctions were detected at various time points during wobbler disease. In detail, a restricted function of complexes I, III, and IV of the mitochondrial electron transport chain and a reduced oxygen consumption rate of complex I were observed on isolated mitochondria of wobbler brain [27]. Dave et al. [28] confirmed these differences and demonstrated their presence even at earlier time points of the disease. In accordance with this, Santoro et al. [29] demonstrated a reduced oxygen consumption rate of complexes I and IV of the mitochondrial electron transport chain as well as a reduced activity of complex I in isolated mitochondria of the cervical part of the spinal cord. Furthermore, decreased activities of respiratory complexes I, II, and III were found in mitochondria of the cervical and partially (complex I only) lumbar spinal cord [30]. Previous studies in SOD1^G93A^ transgenic mice found that oxidative stress leads to fragmentation of the mitochondrial network and ultimately neurodegeneration [19]. This fragmentation was caused by a deregulation of fission and fusion-related enzymes due to increased oxidative stress [20]. Our previous study revealed that increased levels of reactive oxygen species (ROS) are present in the cervical spinal cord of wobbler mice [31]. Here, we aimed to decipher the consequences of increased ROS on mitochondrial network, individual mitochondrial parameters, and mechanisms of mitochondrial dynamics in motor neurons of wobbler mice in order to reveal common parallel pathomechanisms between different ALS models. These findings may open new treatment strategies which are independent of the present genotype and thus beneficial to a larger ALS patient cohort.

## 2. Materials and Methods

### 2.1. Animals

All procedures were conducted under established standards of the German federal state of North Rhine Westphalia, in accordance with the European Communities Council Directive 2010/63/EU on the protection of animals used for scientific purposes. Animal experiments were conducted according to the German animal welfare regulations and approved by the local authorities (registration number Az. 84-02.04.2017.A085). The used mouse strain is C57BL/Fa carrying the wobbler point mutation in the *VPS54* gene. Breeding and genotyping were carried out as described previously [22]. The mice were kept in a 12 h night/day cycle and had access to food and water *ad libitum*. Cervical spinal cord tissues from WT and WR animals were collected at the age of p20 and p40 and used for further experiments. All experiments were exclusively carried out with 3-10 homozygous WT or WR mice. Both genders were used. Heterozygous animals were used for breeding.

### 2.2. Motor Neuron Enriched Dissociated Cell Culture of the Ventral Horn

The protocol for the cultivation of dissociated spinal cord cell cultures was performed as described before [26]. In brief, homozygous mice were decapitated at the age of p40 and spinal cords were removed. After removing meninges and separating the anterior horns, tissues were cut into small fragments and digested with 36 U/ml papain (#LS003119, Cell Systems, Germany) isolation medium [0.5 mM GlutaMax (#35050061, Thermo Fisher Scientific, Germany), 100 U/ml Penicillin/Streptomycin (#P4333, Merck, Germany), and 2% B27 supplement (#17504044, Thermo Fisher Scientific, Germany) in Hibernate A (#A1247501, Thermo Fisher Scientific, Germany)] for 10 min at 37°C. After trituration of the tissue, the isolated cells were separated by density gradient centrifugation on an OptiPrep (#1114542, Progen, Germany) density gradient according to Zwilling et al. [26]. The cells from fractions 2 and 3 were pelletized by centrifugation (244 g for 6 min at 10°C) and plated in motor neuron feeding medium [30% C2C12 myocyte-conditioned medium [32], 0.5 mM glutamine (#G7513, Merck, Germany), 100 U/ml Penicillin/Streptomycin, 2% B27 supplement, 125 mM cAMP (#A6885, Merck, Germany), 1 ng/ml BDNF (#CYT-207, Prospec, Israel), and 0.1 ng/ml GDNF (#CYT-305, Prospec, Israel) in Neurobasal A (#10888022, Thermo Fisher Scientific, Germany)] at a density of 70.000 cells per well onto poly-D-lysine- (50 *μ*g/ml, #P7280, Merck, Germany) coated high precision glass cover slides (12 mm). The cells were cultivated in vitro for 10 days at 37°C and 5% CO_2_ with a medium change after 2 to 3 days.

### 2.3. Immunofluorescence Staining

Immunofluorescence staining have been performed in dissociated motor neuronal enriched cultures. After 10 days *in vitro*, cells were incubated in neuron feeding medium containing CellTracker (10 *μ*M, #C2925, Thermo Fisher Scientific, Germany) and MitoTracker (100 nM, #M22426, Thermo Fisher Scientific, Germany) for 40 min under normal incubation conditions. Subsequently, the cells were fixed with 4% paraformaldehyde, and nuclei were stained with DAPI (#D9542, Merck, Germany). To investigate the mitochondrial network of motor neurons from WT and WR, cultures were imaged with a superresolution microscope with structured illumination (Zeiss Elyra PS.1 LSM880, Carl Zeiss Microscopy GmbH, Germany) in combination with a 63x oil immersion objective (Plan-Apochromat 63x/1.4 Oil DIC, Carl Zeiss Microscopy GmbH, Germany) equipped with respective filter sets. We have focused here on mitochondria in the perinuclear and soma region as these contain the largest number of mitochondria and are most critical in terms of mitochondrial degradation, making them best suited for a description of the network. Imaris 9.2.1 (Oxford Instruments, UK) surface and spot function were used for the evaluation of parameters describing the mitochondrial network precisely. First, the motor neuron was manually marked, and a region of interest was defined. Outside this region of interest, all voxels were set to 0. This guaranteed that results were not influenced by cells in the surrounding area. The spot function forms spheres around the individual signals, while the surface function maps the structure of the mitochondrial chains. For the surface function, we set a threshold value of 150 and for the spot function a threshold value of 200 and the quality filter type. At least 50 motor neuronal cells were measured for each genotype. All obtained parameters were quantitatively evaluated using GraphPad Prism 7 software (GraphPad Software, USA). Data are presented as the mean values ± SEM. The Kolmogorov-Smirnov normality test was used to confirm normal distribution. Student's *t*-test was performed for significance testing between the two genotype groups, and values with *p* < 0.05 were considered to be significant.

### 2.4. Transmission Electron Microscopy

After describing the mitochondrial network in the soma of motor neurons with aid of immunofluorescence staining, the question arose if the network structure is reflected in abnormal individual mitochondria. To close this gap, TEM studies were performed. The embedding protocol was based on Krause et al.'s study [33]. Mice were anaesthetized with Ketamine (100 mg/kg) and Xylazine (10 mg/kg) and transcardially perfused with 2.5% glutaraldehyde (#G5882, Merck, Germany) in phosphate buffer (PB). After incubation of the tissue in Dalton solution [1 g OsO4 (#19134, Electron Microscopy Sciences, Belgium) solved in 100 ml 5% potassium dichromate solution (#7953, Roth, Germany)] for 2 h, tissue was washed with PB. Next, specimens were dehydrated through an ascending ethanol series, starting with 50%-ethanol, followed by incubation in 70% ethanol, 1% uranyl acetate (#21447, Polyscience Inc., England) and 1% phosphotungstic acid (#455970, Merck, Germany) solution overnight at 4°C. The next day, dehydration continued with an ascending ethanol series (80-100%). The specimens were carefully transferred into epoxy resin. This was accomplished by first incubating the tissue in propylene oxide (#807027, Merck, Germany), followed by an ascending series of propylene oxide and EPON mixtures. This embedding procedure started with propylene oxide/EPON in a 3 : 1 ratio, followed by a 1 : 1 ratio, and ended with a 1 : 3 ratio. Finally, specimens were penetrated by pure EPON overnight at 20°C. On the third day of embedding, EPON was renewed. After all, EPON embedded specimens were allowed to polymerize at 60°C for two days. EPON consists of glycidether (#21045.02, Serva, Germany), methylnadic anhydride (#29452.02, Serva, Germany), 2-dodecenylsuccinic acid anhydride (#20755.01, Serva, Germany), and 2,4,6-tris(dimethylaminomethyl)phenol (#36975.01, Serva, Germany) in a 5.4 : 3.8 : 1.84 : 1 mixture. Ultrathin slices (70 nm) were cut with an Ultracut E Reichert-Jung (Leica Microsystems GmbH, Germany) with a DiATOME histo diamond knife (45°, 6 mm, MX559; Diatome AG, Switzerland). Philipps EM 420 (Philips, Netherlands) and ImageJ 1.51 s (National Institutes of Health, USA) were used for the evaluation of single mitochondria in detail as described below.

### 2.5. Analyses of Morphological Parameters

To describe the morphology of mitochondria in the soma of motor neurons, ultrathin sections (70 nm) of the cervical spinal cord were prepared, and the area of the anterior horn was magnified. Overview photographs were taken to identify the motor neurons. Therefore, motor neuron-specific characteristics such as size, shape, nucleus, soma texture, and Nissl bodies were considered. Next, the number of mitochondria within a motoneuronal soma was counted. In the next step, the area around the nucleus was highly magnified and each mitochondrion within the motor neuron was captured. In order to describe the mitochondrial morphology in detail, various parameters were evaluated which have turned out to be proven for describing mitochondria in a previous study [34]. In detail, the following parameters were measured:

- Surface area (in nm^2^)
- Perimeter in 2D (in nm)
- Aspect ratio (major to minor axis)
- Feret's diameter (longest distance in one single mitochondrion)
- Roundness (rated by 4∗area/*π*∗major axis^2^)
- Circularity (rated by 4 *π*∗area/perimeter^2^)
- Circularity value of 100% expressing a perfect circle
- Elongated mitochondria having a circularity value closer to 0

Obtained parameters were quantitatively evaluated using GraphPad Prism 7 software (GraphPad Software, USA). The data are presented as mean values ± SEM. The Kolmogorov-Smirnov normality test was used to confirm normal distribution. Student's *t*-test was performed for significance testing between the two genotype groups, and values with *p* < 0.05 were considered to be significant.

### 2.6. TEM Tomography

During the evaluation of TEM images, the assumption arose that the IMM and crista structure of wobbler mitochondria seems to be altered. To examine these changes in detail, we studied the mitochondria with TEM tomography. First, mice were anaesthetized and transcardially perfused with 2% formaldehyde (#15714-S, Electron Microscopy Sciences, USA), 2.5% glutaraldehyde (#G5882, Merck, Germany), and 2 mM CaCl_2_ in 0.15 mM cacodylate buffer. In the subsequent step, samples were stained with 2% osmium tetroxide and 1.5% potassium ferrocyanide in 0.15 mM cacodylate buffer for 1 h. Further, specimens were treated with 1% thiocarbonohydrazide (#T2137, Merck, Germany) for 25 min, 2% osmium tetroxide (#0972B-6, Polyscience Inc., England) for 30 min, and finally 2% uranyl acetate (#21447, Polyscience Inc., England) overnight at 4°C (each solved in H_2_O). The next day, the samples were stained with 0.66% lead nitrate (#HN32.1, Carl Roth, Germany) in 3 mM aspartic acid (#A9256, Merck, Germany) for 30 min. Next, samples were dehydrated by incubation in an ascending ethanol series starting with 30% ethanol, followed by 50%, 70%, 80%, and 96%, and finally pure ethanol. The samples were then briefly immersed in propylene oxide (#807027, Merck, Germany) and afterwards in a Durcupan (#44610, Merck, Germany)/propylene oxide mixture in a 1 : 2 and subsequent 3 : 1 ratio. Finally, the samples were embedded overnight in pure Durcupan and then polymerized in fresh Durcupan at 60°C for 3 days. Slices (thickness approximately 200 nm) were cut with an Ultracut E Reichert-Jung (Leica Microsystems GmbH, Germany) with a DiATOME histo diamond knife (45°, 6 mm, MX559; Diatome AG, Switzerland). Single axis tilt series were recorded on a JEOL JEM-1400 Plus transmission electron microscope (JEOL, Japan) operating at a 120 kV with a LaB6 filament and equipped with a 4096 × 4096-pixel CMOS camera (TemCam-F416, TVIPS, Germany). Automated image acquisition (16 Bit resolution, 4096 × 4096 pixels, pixel size: 1,213 nm) over an angular range from -60° to 60° with 1° step size was performed using the software serialEM 3.58 (University of Colorado, USA) [35].

### 2.7. 3D Reconstruction of Mitochondria

Image alignment and reconstruction by filtered back projection was carried out using the software package IMOD 4.9.7 [36]. A 3D model was generated by manual segmentation of the reconstructed image stack with the segmentation feature of 3dMod from the IMOD package. On each image plane, individual objects with corresponding contours specific to the mitochondrial structures were assigned and exported as a surface mesh. Small mismatches and failures in the mesh were corrected with the software MeshLab 2020.07 (Institute of Information Science and Technology, Italy), and the final result was rendered by a raytracing algorithm implemented in the software Blender 2.83.2 (Blender Foundation, Netherlands).

### 2.8. qPCR

Since our previous studies revealed an altered mitochondrial network with smaller individual mitochondria, we intended to take a closer look at the fusion- and fission-related mechanisms. To accomplish this, qPCR and Western blotting were performed. Total RNA (tRNA) was extracted from the cervical spinal cord tissue of WT and WR mice at p40 using NucleoSpin miRNA Kit (#740971, Macherey-Nagel, Germany) according to the manufacturer's protocol. cDNA synthesis was performed with a reverse transcription system (#A3500, Promega, USA). Following the manufacturer's protocol, 1 *μ*g tRNA and oligo(dT)15 primer were used. The cDNA was stored at -20°C until use. Standardized quantitative real-time PCR was performed on a CFX96 Real-Time PCR Detection System (Bio-Rad, USA). GoTag qPCR Master Mix (#A6001, Promega, USA) was used with 50 ng cDNA and the corresponding primer sets (0.7 *μ*M each). The following primer sequences were used: *GAPDH*—5′-GGA GAA ACC TGC CAA GTA TGA-3′ (sense) and 5′-TCC TCA GTG TAG CCC AAG A-3′ (antisense), *Mfn1*—5′-AGA CTG TTA ATC AGC TGG CCC-3′ (sense) and 5′-GGT CAT CTC TCA AGA GGG CA-3′ (antisense), *Mfn2*—5′-ATG CTT CCC CTC TCA AGC AC-3′ (sense) and 5′- GCT CTC TTG GAT GTA GGC CC-3′ (antisense), *Opa1*—5′-ACG GGT TGT TGT GGT TGG AG-3′ (sense) and 5′-GTG TCA TCA TCT CGC CGG AC-3′ (antisense), *Oma1*—5′-GGG CAG GGG CAT AAG GAA AT-3′ (sense) and 5′-ACT CAG ACC AAG AAG CAG CC-3′ (antisense), and *Dnm1l*—5′-GTA AGC CCT GAG CCA ATC CA-3′ (sense) and 5′-CTC GAT GTC CTT GGG CTG AT-3′. Melting curves were recorded after each cycle and showed individual PCR products. Expression levels of the genes of interest and the housekeeping genes were measured in triplicate in three independent PCR runs. The collected data were analyzed using the 2^−*ΔΔ*CT^ method [37]. GraphPad Prism 7 software (GraphPad Software, USA) was used for data evaluation. The data are presented as the mean values ± SEM. The Kolmogorov-Smirnov normality test was used to confirm normal distribution. Student's *t*-test was performed for significance testing between the two genotype groups, and values with *p* < 0.05 were considered to be significant.

### 2.9. SDS Gel Electrophoresis and Western Blotting

For Western blotting, proteins from cervical spinal cord were isolated using cell lysis buffer (#9803S, Cell Signaling Technology, USA) supplemented with protease inhibitor (#11697498001, Merck, Germany). To determine protein concentrations, Pierce™ BCA Protein Assay Kit (#23225, Thermo Fisher Scientific, Germany) was used. 50 *μ*g of total protein was separated by SDS gel electrophoresis and transferred to a nitrocellulose membrane. Subsequently, the blots were blocked by incubation in 1% RotiBlock (#A151, Roth, Germany) in phosphate-buffered saline (PBS) for at least 1 h at room temperature. Primary antibodies (Table 1) were incubated overnight at 4°C. HRP-coupled secondary antibodies (Table 1) were incubated for 1 h at room temperature. Finally, Immuno Cruz Luminol Agent (#sc-2048, Santa Cruz Biotechnology, USA) was used for signal detection with an imaging system (ChemiDoc XRS+, BioRad, USA). For arithmetic analysis of the band intensity, ImageJ 1.51s (National Institutes of Health, USA) software was used. Band intensities of interested proteins were normalized to the housekeeper calnexin or actin. Normalized protein levels were compared between different genotypes. Data analyses were performed using GraphPad Prism 7 software (GraphPad Software, USA). The results are presented in bar charts with the respective percentage. Data were reported as normalized means ± SEM. The Kolmogorov-Smirnov normality test was used to confirm normal distribution. Student's *t*-test was performed for significance testing between the two genotype groups, and values with *p* < 0.05 were considered to be significant.

## 3. Results

In numerous studies on neurodegenerative diseases, altered mitochondria were a common phenomenon observed in the affected cells [38–40]. In the pathogenesis of Alzheimer's disease, for example, a reduction in the number and size of mitochondria has been identified as a key step in pathogenesis [41]. Especially in ALS, mitochondria seem to play a crucial role in the degeneration of mitochondrially highly active motor neurons [20]. Based on our recent findings of elevated ROS levels in wobbler mice [31], our interest was focused on motor neuronal mitochondria in this study. The purpose of this study was to reveal mitochondrial abnormalities in motor neurons of wobbler mice and to provide evidence for possible pathological underlying mechanisms.

### 3.1. Disturbed Mitochondrial Network in Wobbler *α*-Motor Neurons

**Figure 1:**
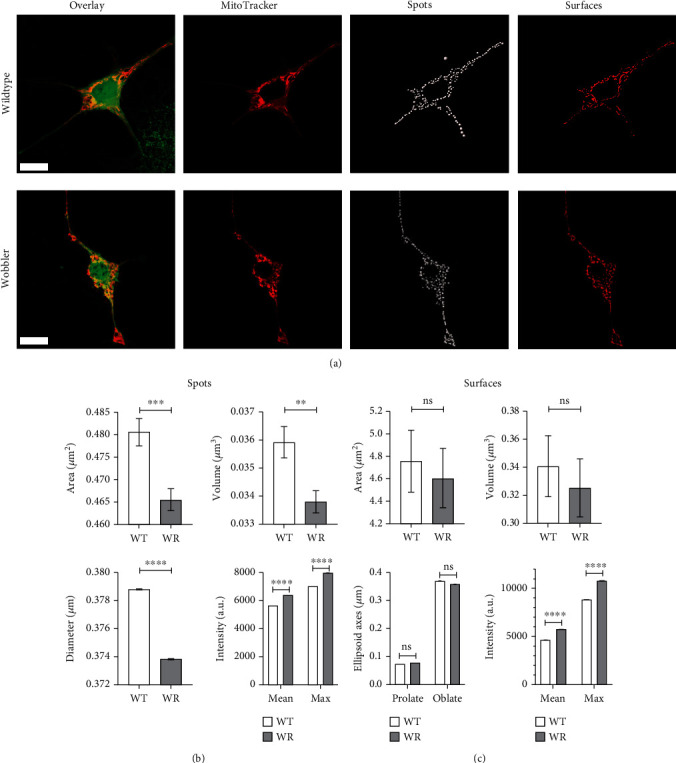
Disturbed mitochondrial network in wobbler *α*-motor neurons. (a) Motor neuron enriched cultures of p40 WT and WR after 10d *in vitro*. Staining was performed with CellTracker (green) and MitoTracker (red). The mitochondrial network was reconstructed and quantitatively analyzed with Imaris 9.2.1 (b) spots and (c) surface function. Due to a significant decrease in area, volume, and diameter of spots as well a slightly diminished surface area and volume, a disturbed, fragmented mitochondrial network is present in wobbler motor neurons. No clear differences could be found in surface ellipsoid axes. Mean and maximum MitoTracker intensity of spots and surfaces are significantly higher in wobbler motor neurons, probably explained by more individual, smaller mitochondria per area. Data are presented as means ± SEM. For significance testing, students t-test was performed. Significant differences are indicated by ns > 0.05, ^∗∗^*p* < 0.01, ^∗∗∗^*p* < 0.001, and ^∗∗∗∗^*p* < 0.0001. Scale bar = 10 *μ*m (a). A total of 50 motoneurons from four independent preparations per genotype were examined. *n*(spots) = 10415-11815; *n*(surfaces) = 1290-1433.

We used dissociated cell cultures and immunofluorescent staining to visualize the mitochondrial network in the soma of motor neurons. Therefore, we cultivated dissociated motor neuronal enriched cultures of wild-type (WT) and wobbler diseased (WR) mice for 10 days. We compared the mitochondrial network of 50 motor neurons from WT and WR of three independent preparations with aid of superresolution microscopy with structured illumination in combination with the analysis software Imaris 9.2.1. We used the spots and surface function to compare the MitoTracker signal and thereby the mitochondrial network (Figure 1(a)). The surface function enables the study of mitochondrial chains and thus the continuity of the mitochondrial network. The spot function rather represents higher local intensities within the network or single mitochondria. Obtained parameters were quantitatively evaluated. The area, volume, and diameter of spots and thus single mitochondria were significantly diminished in homozygous wobbler mice (Figure 1(b)). Surface function showed a trend towards smaller mitochondrial chains in motor neurons from diseased mice, due to a slightly smaller surface area and volume (Figure 1(c)). By evaluating the mitochondrial chains/surfaces, we did not find any clear differences in the ellipsoid axes. But we were able to detect strong differences in MitoTracker signal intensity in the motor neuronal soma. Both the mean and the maximum intensity of MitoTracker signal were significantly increased for the measured wobbler spots and surfaces compared to WT in the region of interest (Figures 1(b) and 1(c)). This could be related to an accumulation of single mitochondria, resulting in stronger MitoTracker signals per area.

### 3.2. Smaller and Irregularly Shaped Mitochondria of Wobbler Mice Motor Neurons

**Figure 2:**
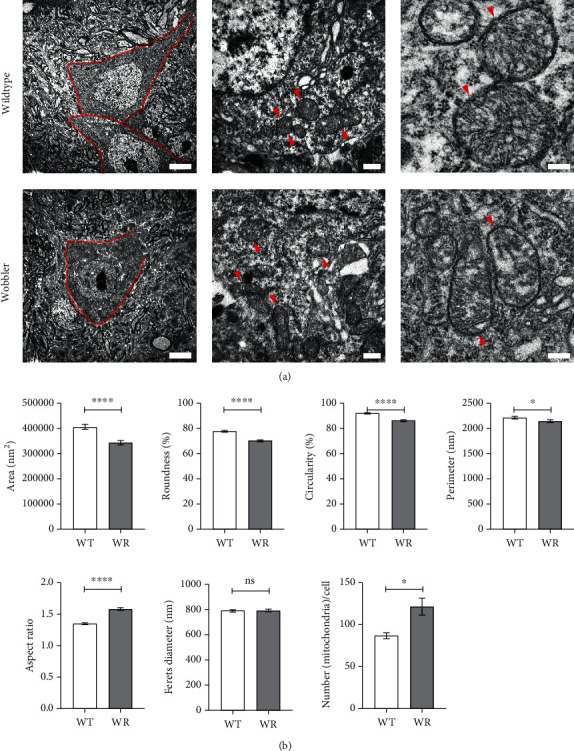
Smaller and irregularly shaped mitochondria of wobbler mice motor neurons at p40. Transmission electron microscopy of cervical spinal cord of wild-type and wobbler mice at p40. (a) Overview images of motor neurons (red border) and magnified single mitochondria (red arrowheads), indicating an altered mitochondrial morphology and degeneration of crista structure in wobbler mice. (b) Measurement of mitochondria with ImageJ revealed a significant decrease in area, roundness, circularity, and perimeter combined with a significantly increased aspect ratio in wobbler mice. No differences in Feret's diameter could be detected. Counting mitochondria demonstrated an increase of mitochondrial number per motoneuron. In summary, smaller, irregularly shaped, and elongated mitochondria are present in wobbler motor neurons at p40. Data are presented as the means ± SEM. For significance testing, Student's *t*-test was performed. Significant differences are indicated by ns > 0.05, ^∗^*p* < 0.05, and ^∗∗∗∗^*p* < 0.0001. Scale bar = 5 *μ*m (left), 500 nm (middle), and 200 nm (right). *N* = 5; *n* = 1066-1092 mitochondria per genotype.

Since our immunofluorescence investigations showed evidence of smaller spots in the mitochondrial network, we aimed to clarify whether this might be reflected in detail by morphologically abnormal mitochondria. Therefore, transmission electron microscopy (TEM) of the cervical spinal cord of WT and WR mice was performed (Figure 2(a)). Quantitative and qualitative analyses of specific characteristics revealed abnormal mitochondrial morphology in motor neurons of WR mice (Figure 2(b)). The analysis revealed that WR mitochondria are significantly smaller than WT mitochondria. In addition, a significant decrease in roundness, circularity, and perimeter was identified in WR mitochondria compared to WT mitochondria. Aspect ratio was significantly increased in WR compared to wild-type littermates. No clear differences could be found in Feret's diameter. Counting mitochondria revealed significant more mitochondria per motor neuron in wobbler mice. Altogether, smaller and elongated mitochondria were detected in motor neurons of wobbler mice at the stable phase of the disease (p40) compared to wild-type mice using TEM. Qualitative observation revealed internal vacuolization, misfolded inner mitochondrial membrane (IMM), and altered crista structure in most mitochondria in WR motor neurons, indicating degeneration of these organelles. In contrast, we observed larger mitochondria with intact IMM in motor neurons at the time of the first appearance of symptoms (p20) in the wobbler mice compared to wild-type (Figure S1).

### 3.3. 3D Visualization of the Mitochondrial Crista Structure

**Figure 3:**
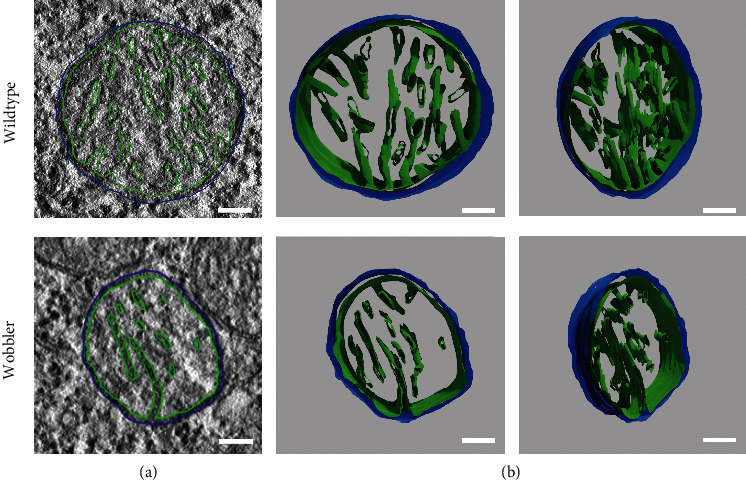
Three-dimensional visualization of the mitochondrial crista structure. TEM tomography of cervical spinal cord of wild-type and wobbler mice. (a) Exemplary image of a recorded plane of TEM tomography with reconstruction of mitochondrial membranes. Blue line represents the modulation of the outer mitochondrial membrane (OMM), while the green line modulates the inner mitochondrial membrane (IMM). (b) Front view and tilted view of the finally meshed 3D models of WT and WR mitochondria. Models imply a misfolded, smaller IMM and a reduction in cristae as well as crista junctions. *N* = 2. Scale bar = 100 nm.

Since evidence of a misfolded IMM was considered by TEM in p40 motor neurons of mitochondria, we visualized this in more detail by TEM tomography. The aim was to reveal possible differences in crista junctions or crista shape of the inner mitochondrial membrane. Motor neurons were identified by their specific characteristics, and mitochondria were selected analogous to our prior TEM investigations. TEM tomography supported the assumption derived from our TEM studies suggesting a degenerated and misfolded IMM as shown in Figure 3. In addition to reduced mitochondrial area and its irregular shape in WR motor neurons observed by TEM, TEM tomography revealed strong indications for a reduction of the IMM area in WR motor neurons. Moreover, the number of cristae and thus crista junctions per mitochondrion might be reduced compared to wild-type mitochondria (Figure 3). Additional movie files show a reconstruction of the z-stack of a wild-type and wobbler mitochondrion (Movie S1) and videos of the 3-dimensional model of these mitochondria (Movie S2).

### 3.4. Abnormal Fission-Related Proteins in the Cervical Wobbler Spinal Cord

**Figure 4:**
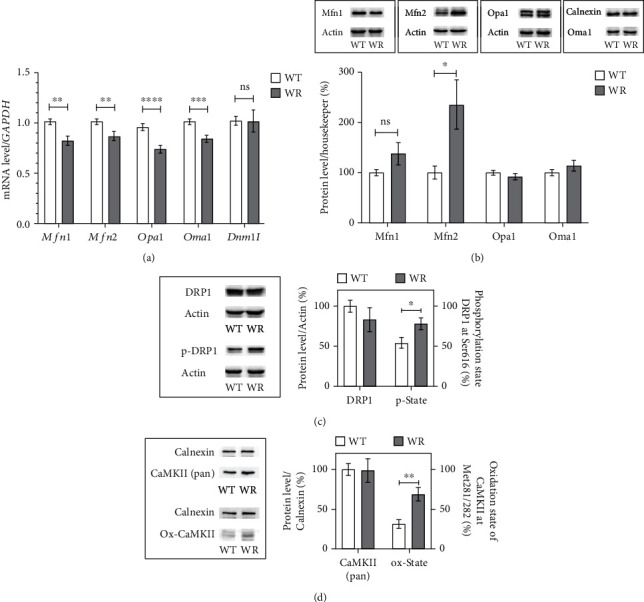
Abnormal fission-related proteins in cervical wobbler spinal cord at p40. (a) mRNA expression levels of *Mfn1*, *Mfn2*, *Opa1*, *Oma1*, and *Dnm1l* from the stable phase of wild-type (WT) and wobbler (WR) spinal cords were investigated by qPCR. mRNA levels were significantly reduced in WR except *Dnm1l*. For relative quantification, the 2^−∆∆Ct^ method was conducted using *GAPDH* for normalization. *N* = 7-11 per genotype. (b) Exemplary Western blots of Mfn1 (≈85 kDa), Mfn2 (≈85 kDa), Opa1 (≈100 kDa), and Oma1 (≈50 kDa) in the cervical spinal cord of p40 WT and WR. Actin (≈45 kDa) and calnexin (≈90 kDa) were used as control proteins. Bar charts represent the semiquantitative analysis of protein expression levels. Western blots revealed unchanged expression of Mfn1, Opa1, and Oma1 as well as significantly increased expression of Mfn2 in the cervical spinal cord of wobbler mice. *N* = 8-10 per genotype. (c) Exemplary Western blots of Drp1 (≈85 kDa), p-Drp1 (Ser616; ≈85 kDa), and actin (≈45 kDa) as control protein. Analysis of band intensity is presented in bar charts. Total amount of Drp1 does not differ between the two genotypes; Drp1 is significant more often phosphorylated at Ser616 in cervical spinal cords of wobbler mice. *N* = 9 per genotype. (d) Exemplary Western blots of CaMKII (≈55 kDa) and oxidized Ox-CaMKII (Met281/282; ≈55 kDa) in combination with calnexin (≈90 kDa) as control protein from wild-type and wobbler cervical spinal cords. Analysis of band intensity is presented in bar charts and showed equal CaMKII levels; thus, the proportion of oxidized CaMKII at Met281/282 is significantly increased in wobbler spinal cords compared to wild-type. *N* = 7 per genotype. All data are presented as the mean values ± SEM, and Student's *t*-test was performed for significance testing between WT and WR. Values with *p* < 0.05 were considered to be significant. Significant differences are indicated by ns > 0.05, ^∗^*p* < 0.05, ^∗∗^*p* < 0.01, ^∗∗∗^*p* < 0.001, and ^∗∗∗∗^*p* < 0.0001.

Since our data demonstrated an abnormal mitochondrial morphology in motor neurons of the cervical spinal cord of wobbler mice, the question arose whether altered mitochondrial dynamics are present in wobbler mice at the stable phase of the disease. Here, we found that all investigated mRNA levels of fusion-related proteins *Mfn1* and *Mfn2*, *Opa1*, and *Oma1* were significantly reduced in cervical spinal cord of WR mice (Figure 4(a)). In order to verify these findings on a protein level, Western blots were performed (Figure 4(b)). It was not possible to confirm the expression differences found on mRNA level. On the contrary, Mfn2 protein expression was found to be significantly increased in WR motor neurons. On the site of fission related proteins, we took a closer look at the expression of the gene *Dnm1l* and the corresponding Drp1 protein. The expression of total Drp1 did not differ between WT and WR, neither on mRNA nor on protein level (Figure 4(c)). We further investigated important mechanisms influencing the activity of Drp1. In order to do so, we first investigated the phosphorylation status of the Drp1 at Ser616, as this phosphorylation is associated with increased Drp1 activity. Our investigations discovered that a significant larger proportion of Drp1 was phosphorylated at Ser616 in wobbler spinal cord in contrast to WT (Figure 4(c)). Furthermore, we could show that the Ca2+/calmodulin kinase (CaMKII), an important posttranslational regulator of Drp1, was significantly more abundant in its oxidized and thus activated form in WR motor neurons compared to WT (Figure 4(d)).

## 4. Discussion

It is well known that oxidative stress in motor neurons leads to massive impairments and degenerative changes [42, 43]. In various neurodegenerative diseases such as Parkinson's disease, Alzheimer's disease, Huntington's disease, autosomal-dominant optic neuropathy, and even some human ALS cases, several defects in mitochondrial function, dynamics, and ROS production, leading to oxidative stress and thus degeneration, have been identified in causal relations [38]. This underlines the importance of healthy mitochondria for the maintenance of neuronal structures. Our previous studies have been able to uncover strongly increased ROS levels and indications for defects in antioxidant capacity in the spinal cord of wobbler mice, an ALS animal model, at the stable phase of the disease [26, 31]. The purpose of this study was to reveal morphological differences in mitochondrial architecture and network. It is questionable whether impaired mitochondria are additionally leading to high ROS values or if impaired mitochondrial function and morphology are a consequence of increased ROS levels. Based on this question, we aimed to find possible primary causes of mitochondrial changes that would explain the previously found elevated ROS levels. Finally, this work should clarify whether there are common pathomechanisms with other ALS models and human ALS cases. This would possibly provide new therapeutic targets from which a larger cohort of patients could benefit in the future.

Mitochondria provide the energy supply needed by the machinery of every living cell. The majority of mitochondria are found in the cell soma. Furthermore, processes responsible for the degradation of damaged mitochondria by autolysosomes also predominantly take place in this area. For this reason, the cell soma is particularly important for the detection of increased mitochondrial degradation [44], and we therefore focused on the characterization of mitochondria in this region.

In this study, we showed that the mitochondrial network of wobbler motor neurons from p40 mice is split into more individual, smaller, single components in comparison to the wild-type mitochondrial network. This is represented by a similar size of the reconstructed surfaces and a reduction in the spot size. We assume that these individual, smaller components are accumulating, explaining the strong increased MitoTracker signal in our immunofluorescence investigations. We were also able to show on ultrastructural level smaller and elongated mitochondria due to a decrease in perimeter and size as well as an increase in aspect ratio and number of mitochondria per motoneuron in stable diseased mice. This is combined with an irregular shaped morphology of mitochondria through a diminished roundness of wobbler mitochondria in motor neurons compared to wild-type. By 3D reconstruction of a mitochondrial section with TEM tomography, we confirmed further endorsement for these differences and additionally identified strong indications for a misfolded, smaller inner mitochondrial membrane, and reduced cristae in combination with less crista junctions in WR motor neurons in comparison to wild-type. In contrast to the late diseased stage in wobbler mice at p40, the occurrence of the first ALS typical symptoms at p20 went along with larger and rounder mitochondria in the motor neurons of the cervical cord without signs of degeneration of the IMM. Others described the mechanism of fusion with a neighboring healthy mitochondrion as a process attempting to mitigate an existing damage, thus enabling to mix mitochondrial DNA, proteins, lipids, and metabolites [45–47]. However, in case the mitochondrial membrane potential cannot be maintained and the organelle is severely damaged, a mitochondrial fusion process cannot occur to maintain mitochondrial integrity. Thus, the cell implies mitochondrial fission processes to generate small mitochondria that can be more easily degraded [48–51]. Our results indicate the existence of such a process within the motor neurons of wobbler mice. Here, the assumption arises that at stage p20, mitochondria are still undergoing fusion to minimize damage that has occurred. Since we did not detect increased ROS levels in the cervical spinal cord in a previous study at p20 [31], another stressor must be involved as a trigger of mitochondrial damage. Another hypothesis would be that more ROS are already produced in motor neurons at p20 compared with wild-type mice, while antioxidant mechanisms still function sufficiently to counteract this imbalance. Thus, further studies are needed to identify or verify the actual stressor that leads to mitochondrial damage early in the disease. This stressor will likely still be present after p20 and continue to damage mitochondria, so fusion will not mitigate mitochondrial damage over the long term. The mitochondria cannot maintain their membrane potential, and the cells are more likely to undergo fission, as we see in the motor neurons of wobbler mice at stage p40. Furthermore, it is known that in diseases associated with decreased mitochondrial respiratory chain function, as it appears to be the case in ALS [20] and wobbler mice [27–30], decreased mitochondrial membrane potential is associated with an increase in ROS production at mitochondria [28, 52].

A fragmentation of the mitochondrial network to smaller single components has already been described in many neurodegenerative diseases including some subtypes of ALS [38, 53]. Ultimately, these cases could be attributed to an imbalance in fusion-fission activity. For this reason, we initially suspected that a restricted fusion activity or increased fission activity could be causative for the fragmentation at the stable phase of the wobbler disease. Therefore, we investigated the expression of fusion and fission-related proteins. Here, we could not find any differences in the expression of fusion-related proteins that would explain the described morphology of the mitochondria and the deconstructed mitochondrial network in the motor neurons of diseased animals at p40. Strikingly, the expression of the fusion-related protein Mfn2 was increased in the cervical spinal cord of wobbler animals. Previous studies could only attribute fragmentation of the mitochondrial network to a downregulation of Mfn2 [54]. In these cases, fragmentation subsequently led to increased glutamate-induced excitotoxicity and neuronal cell death. On the contrary, it has been shown that upregulation of Mfn2, as we could show in wobbler tissue, has a protective effect against these events [55]. In particular, considering that glutamate-induced excitotoxicity plays a central role in the pathophysiology of ALS [56], Mfn2 upregulation could be interpreted as a compensatory mechanism for the cell to protect itself from glutamate-induced excitotoxicity. Interestingly, an overexpression of Mfn1 has been found in *C9Orf72*-ALS/FTD patient fibroblasts in combination with mitochondrial fragmentation as well [57]. There is some debate whether this is a compensatory mechanism [20]. More detailed studies are needed to confirm this mechanism, as it is not fully understood to date.

Studies in neurodegenerative Parkinson's disease reveal an overexpression of the fission related protein Drp1. This caused segmentation of the mitochondrial network into smaller components and led to neuronal degeneration [58]. Although the mitochondrial network described in these Parkinson cases is very similar to the mitochondrial network found in the motor neurons of our wobbler mice, we could not confirm a Drp1 dysregulation in our study. Our analyses of the fission-related protein revealed no abnormalities at the mRNA or at the protein level. For this reason, dysregulated mitochondrial fission initially seemed to be unsuitable as a reason for the segmentation.

Increased oxidative stress leads to numerous damages in the cell, some of which can further multiply the oxidative stress [59]. An elevated ROS level in the spinal cord of wobbler mice could lead to DNA damage, lipid oxidation, protein oxidation and aggregation, induction of inflammatory processes, excitotoxicity, reduction in the efficiency of cellular process, and apoptosis [20, 60, 61]. Mitochondrial DNA is particularly sensitive to ROS since mitochondria have limited DNA repair mechanisms [20]. Elevated ROS levels are frequently described and discussed in the context of ALS (reviewed in 63). Even in biofluid samples and postmortem tissue biopsies from ALS patients [62, 63], elevated biomarkers for oxidative stress have been detected. Additional consequences of oxidative stress were studied in SOD1^G93A^ transgenic mice, a mouse model for familiar form of ALS, which are summarized in Barber and Shaw's study [64]. In this context, it must be emphasized that oxidative stress leads to aggregation of SOD1 [65], which induces mitochondrial dysfunction [66]. This finally ends in a self-reinforcing process with fatal consequences, as mitochondrial dysfunction further promotes free radical production and thus misfolding of SOD1 [20].

However, it is known that many kinases and proteases carry an oxidizable cysteine residue acting as a sensor for activation, as in case of Ca2+/calmodulin kinase II (CaMKII) [67]. Here, we show that the expression of CaMKII protein does not differ overall between WT and WR, although the proportion shifted significantly towards the oxidized form (Ox-CaMKII) in homozygous wobbler mice in comparison to wild-type animals. Recent studies in triple negative breast cancer cells have shown that oxidation of CaMKII to Ox-CaMKII directly leads to increased phosphorylation of Drp1 at the Ser616. This breast cancer study further revealed that CaMKII is activated by all kind of ROS, especially superoxide anions [68]. In this context, it is interesting to note that impaired functions of complexes I and III of the mitochondrial electron transport chain, as present in the cervical spinal cord of wobbler mice, lead to elevated superoxide levels by electron efflux and spontaneous oxidation of molecular oxygen [28]. In addition to mitochondrial dysfunction, increased activation of microglial cells may increase the expression of NADPH oxidase, which physiologically produces further superoxide anion [13, 64]. Against this background, it is interesting that Dahlke et al. [23] also observed microglial activation in the cervical spinal cord of wobbler mice, probably promoting NADPH expression and thus superoxide anion production. We assume that impaired mitochondrial function in combination with activated microglia causes an increase in superoxide anion production, leading to oxidation of CaMKII at the stable phase of the disease in wobbler mice.

Crucial for the cell are the consequences of CaMKII activation. Calcium-independent activation of CaMKII by methionine oxidation leads to increased fission activity, as it is associated with phosphorylation of Drp1 at the Ser616 and thus increased recruitment of Drp1 to the mitochondrial membrane and fission of mitochondria [69]. Based on our results, the mechanism of ROS-regulated, Ox-CaMKII-dependent Drp1 activation may also play a role in wobbler motor neurons. We showed that Drp1 is significantly more frequently phosphorylated at Ser616 in the cervical spinal cord of diseased animals compared to WT. This could finally explain an increased fission of mitochondria in wobbler motor neurons, ultimately leading to fragmentation of the mitochondrial network into more individual, smaller components. In studies on myocardial ischemia, Drp1-dependent fragmentation of the mitochondrial network was also observed [70]. They discovered that Drp1-dependent fragmentation of the mitochondria increased mitochondrial ROS production. The increased ROS production then further increases mitochondrial fragmentation in terms of a vicious circle [71, 72]. Given our findings of fragmented mitochondria due to Drp1 activation in motor neurons and increased ROS in the spinal cord of wobbler mice at p40, this mechanism might also be present in the wobbler animal model. Fragmented mitochondria have immense consequences for cell physiology. Studies show that Bax and Bak, both proapoptotic molecules, interact with activated Drp1 and thereby initiate apoptosis. Fragmented mitochondria are therefore an early indicator for cell death [40]. In cultured motor neurons of mutant SOD1^G93A^ mice, mitochondrial fragmentation was also observed and directly linked to reduced axonal transport and neurite length and even increased cell death [73]. It is further suggested that fragmented mitochondria are linked to lower ATP levels, reduced mitochondrial membrane potential, and energetically poorer function [20, 74].

Nevertheless, it is still difficult to decide whether increased ROS levels cause fission, leading to dysfunctional mitochondria, or whether dysfunctional mitochondria promote ROS production, leading to increased fission. The outcome of both events is the same. However, since we already detect abnormal mitochondrial morphology at p20 without detecting elevated ROS levels, mitochondrial damage not primarily triggered by oxidative stress can be assumed. Thus, oxidative stress does not seem to be the main factor for motor neuron damage at an early stage of the disease, but a consequence of mitochondrial dysfunction at later stages of wobbler disease. Another point confirming mitochondria as the reinforcing factor of mitochondrial fission in p40 wobbler animals is the fact that impaired mitochondria are the main producers of superoxide anions, which are most important for Ox-CaMKII-dependent Drp1 activation. Moreover, the purpose of Drp1 activation remains partially unclear, as it continuously drives the cell into an apoptotic metabolic state. One possible explanation represents an automechanism by which the cell shuts itself down to prevent a catastrophic event. Another explanation could be that fragmentation causes the degradation of defective mitochondria so that the cell maintains healthy and efficient mitochondrial structures [75]. Further, it was found that Mfn1 or Mfn2 overexpression is able to counteract mitochondrial fragmentation and even attenuate cell death [76]. This would support the hypothesis that increased expression of Mfn2, as we have demonstrated in our study in wobbler spinal cord, is a possible attempt to provide a rescue mechanism of motor neurons from cell death. However, further studies on fission and fusion processes in regard to the interplay between ROS, Drp1, and Mfn2 in motor neurons are needed to gain a full understanding of the underlying mechanism and its potential on motor neuronal degeneration.

## 5. Conclusions

**Figure 5:**
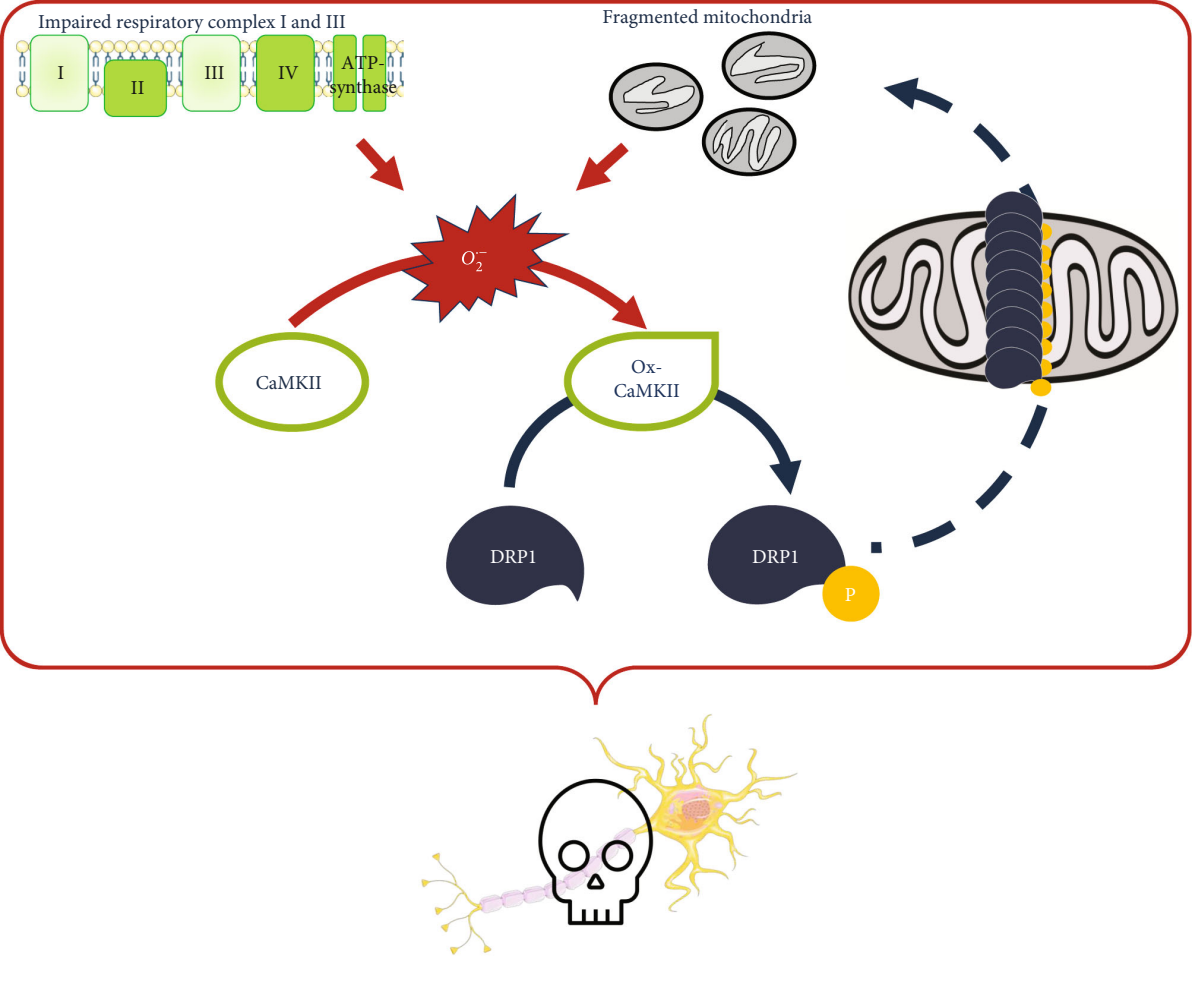
Proposed mechanism of motor neuronal cell death in wobbler mice. An impaired function of complexes I and III of the mitochondrial respiratory chain leads to an increase in superoxide anions. Increased superoxide anion levels cause an oxidation and thus calcium-independent activation of CaMKII. Ox-CaMKII in turn stimulates phosphorylation of Drp1 at Ser616, which recruits it to the mitochondrial membrane and causes enhanced mitochondrial fission. This disruption between fusion and fission balance promotes fragmentation of the mitochondrial network, resulting in increased production of reactive oxygen species. This is likely to trigger a nonreversible process that leads to fragmented and dysfunctional mitochondria, resulting in a self-reinforcing vicious circle that promotes degeneration of motor neurons in wobbler mice.

The present study focused on the morphological analysis of the mitochondrial network and individual mitochondria in motor neurons of wobbler mice, an ALS animal model, in context of previous studies discovering elevated ROS levels in wobbler spinal cords at the stable phase of the disease (p40). We were able to identify several pathologies in wobbler motor neurons, like a fragmented mitochondrial network, consisting of more individual, accumulated mitochondria. We suspect that a reduced function of complexes I and III leads to an increase in superoxide anions, which is maybe further enhanced by activated microglia. Increased superoxide anion levels cause an oxidation and thus calcium-independent activation of CaMKII. Ox-CaMKII in turn stimulates phosphorylation of Drp1 at Ser616, which recruits it to the mitochondrial membrane and causes enhanced mitochondrial fission. Finally, this disrupts the balance between fusion and fission and promotes fragmentation of the mitochondrial network, resulting in increased production of reactive oxygen species (Figure 5). Under physiological circumstances, fission ensures a depletion of dysfunctional mitochondria and maintaining homeostasis. But in context of some ALS cases and probably wobbler disease, mitochondrial fission takes over. This is likely to trigger a nonreversible process that enhances fragmented and dysfunctional mitochondria, resulting in a self-reinforcing vicious circle that additionally promotes degeneration. Nowadays, treatment of ALS patients with Edaravone, the first antioxidant compound for the treatment of ALS that counteracts the process of oxidative stress, reveals promising results, prolonging life by several months. However, since we observe signs of damage in motor neuronal mitochondria at an earlier stage of the disease without evidence of oxidative stress, the search for causative factors should continue. Furthermore, no curative therapeutic approach has been found to date, so further studies are required to cure this fatal disease one day.

## Figures and Tables

**Table 1:** Primary and secondary antibodies used for Western blotting.

Antibody	Dilution	Order number
Anti-MFN1 mouse monoclonal IgG antibody	1 : 100 in Roti-TBS (#1060.1, Roth, Germany)	#sc-166644, Santa Cruz, USA
Anti-MFN2 mouse polyclonal IgG antibody	1 : 100 in Roti-TBS (#1060.1, Roth, Germany)	#ARP89255_P050, Aviva systems biology, USA
Anti-OPA1 mouse monoclonal IgG antibody	1 : 100 in Roti-TBS (#1060.1, Roth, Germany)	#sc-393296, Santa Cruz, USA
Anti-OMA1 mouse monoclonal IgG antibody	1 : 100 in Roti-TBS (#1060.1, Roth, Germany)	#sc-515788, Santa Cruz, USA
Anti-Drp1 mouse monoclonal IgG antibody	1 : 500 in Roti-TBS-T (#1061.1, Roth, Germany)	#14647, Cell Signaling, USA
Anti-pDrp1 rabbit monoclonal IgG antibody	1 : 500 in Roti-TBS-T (#1061.1, Roth, Germany)	#4494, Cell Signaling, USA
Anti-CaMKII (pan) rabbit polyclonal antibody	1 : 500 in Roti-TBS-T (#1061.1, Roth, Germany)	#3362, Cell Signaling, USA
Anti-ox-CaMKII rabbit polyclonal antibody	1 : 500 in Roti-TBS-T (#1061.1, Roth, Germany)	#07-1387 Merck, Germany
Anti-calnexin rabbit polyclonal IgG antibody	1 : 200 in Roti-TBS (#1060.1, Roth, Germany)	#sc-11397, Santa Cruz, USA
Anti-actin rabbit polyclonal IgG antibody	1 : 1000 in Roti-TBS (#1060.1, Roth, Germany)	#A2668; Merck, Germany
Anti-rabbit goat horseradish-peroxidase-conjugated antibody	1 : 10.000 in Roti-TBS (#1060.1, Roth, Germany)	#sc-2054, Santa Cruz, USA
Anti-mouse donkey antibody	1 : 10.000 in Roti-TBS (#1060.1, Roth, Germany)	#sc-2314, Santa Cruz, USA

## Data Availability

All data generated in this study are published in this article and its supplementary files.

## Supplementary Materials

Supplementary Materials Figure S1 (separate file). Larger and Rounder Mitochondria of Wobbler Mice Motor Neurons at p20. Transmission electron microscopy of cervical spinal cord of wild-type and wobbler mice at p20. (A) Overview images of motor neurons (red border) and magnified single mitochondria (red arrowheads), indicating an altered mitochondrial morphology in wobbler mice. (B) Measurement of mitochondria with ImageJ revealed a significant increase in area, roundness, circularity, perimeter, and Ferrets diameter combined with a significantly decreased aspect ratio in wobbler mice. Counting mitochondria demonstrated no significant alteration in mitochondrial number per motoneuron. In summary, larger and rounder mitochondria are present in wobbler motor neurons at p20. Data are presented as the means ± SEM. For significance testing, Student's *t*-test was performed. Significant differences are indicated by ns > 0.05, ^∗^*p* < 0.05, ^∗∗^*p* < 0.01, and ^∗∗∗∗^*p* < 0.0001. Scale bar = 5 *μ*m (left), 500 nm (middle), and 200 nm (right). *N* = 3; *n* = 750 mitochondria per genotype. Movie S1 (separate file). TEM tomography of motor neuronal mitochondria of wild-type and wobbler mice. Exemplary video of different planes of reconstructed z-stack of a wild-type and a wobbler mitochondrion. Scale bar = 100 nm. Movie S2 (separate file). Three-dimensional model of motor neuronal wild-type and wobbler mitochondrion. Image alignment and reconstruction by filtered back projection were carried out using the software package IMOD 4.9.7. A 3D model was generated by manual segmentation of the reconstructed image stack with the segmentation feature of 3dMod from the IMOD package. On each image plane, individual objects with corresponding contours specific to the mitochondrial structures were assigned and exported as a surface mesh. Small mismatches and failures in the mesh were corrected with the software MeshLab 2020.07 (Institute of Information Science and Technology, Italy), and the final result was rendered by a raytracing algorithm implemented in the software Blender 2.83.2 (Blender Foundation, Netherlands).
