# Supplementary material for: Increased ROS-Dependent Fission of Mitochondria Causes Abnormal Morphology of the Cell Powerhouses in a Murine Model of Amyotrophic Lateral Sclerosis
Source: Oxid Med Cell Longev. 2021 Oct 14;2021:6924251. doi: 10.1155/2021/6924251 (PMC8531774; doi:10.1155/2021/6924251)
Supplement: Supplementary Materials — Figure S1 (separate file). Larger and Rounder Mitochondria of Wobbler Mice Motor Neurons at p20. Transmission electron microscopy of cervical spinal cord of wild-type and wobbler mice at p20. (A) Overview images of motor neurons (red border) and magnified single mitochondria (red arrowheads), indicating an altered mitochondrial morphology in wobbler mice. (B) Measurement of mitochondria with ImageJ revealed a significant increase in area, roundness, circularity, perimeter, and Ferrets diameter combined with a significantly decreased aspect ratio in wobbler mice. Counting mitochondria demonstrated no significant alteration in mitochondrial number per motoneuron. In summary, larger and rounder mitochondria are present in wobbler motor neurons at p20. Data are presented as the means ± SEM. For significance testing, Student's t-test was performed. Significant differences are indicated by ns > 0.05, ∗p < 0.05, ∗∗p < 0.01, and ∗∗∗∗p < 0.0001. Scale bar = 5 μm (left), 500 nm (middle), and 200 nm (right). N = 3; n = 750 mitochondria per genotype. Movie S1 (separate file). TEM tomography of motor neuronal mitochondria of wild-type and wobbler mice. Exemplary video of different planes of reconstructed z-stack of a wild-type and a wobbler mitochondrion. Scale bar = 100 nm. Movie S2 (separate file). Three-dimensional model of motor neuronal wild-type and wobbler mitochondrion. Image alignment and reconstruction by filtered back projection were carried out using the software package IMOD 4.9.7. A 3D model was generated by manual segmentation of the reconstructed image stack with the segmentation feature of 3dMod from the IMOD package. On each image plane, individual objects with corresponding contours specific to the mitochondrial structures were assigned and exported as a surface mesh. Small mismatches and failures in the mesh were corrected with the software MeshLab 2020.07 (Institute of Information Science and Technology, Italy), and the final result was rendered by a raytrac [file 6924251.f1.zip › Supplement 2.pdf]

Oxidative Medicine and Cellular Longevity

Increased ROS-Dependent Fission of Mitochondria Causes Abnormal Morphology of The Cells Powerhouses in a Murine Model of Amyotrophic Lateral Sclerosis

Jan Stein[1], Bernd Walkenfort[2], Hilal Cihankaya[1], Mike Hasenberg[2], Verian Bader[3], Konstanze F. Winklhofer[3], Pascal Röderer[1], Johann Matschke[4], Carsten Theiss[1], and Veronika Matschke[1]*

[1] Department of Cytology, Institute of Anatomy, Medical Faculty, Ruhr University Bochum, D-44801 Bochum, Germany

[2] Electron Microscopy Unit, Imaging Center Essen, Medical Faculty of the University of Duisburg-Essen, D-45147 Essen, Germany

[3] Department of Molecular Cell Biology, Institute of Biochemistry and Pathobiochemistry, Medical Faculty, Ruhr University Bochum, D-44801 Bochum, Germany

[4] Institute of Cell Biology (Cancer Research), University Hospital Essen, University of Duisburg-Essen, D-45147 Essen, Germany

Supplementary Materials

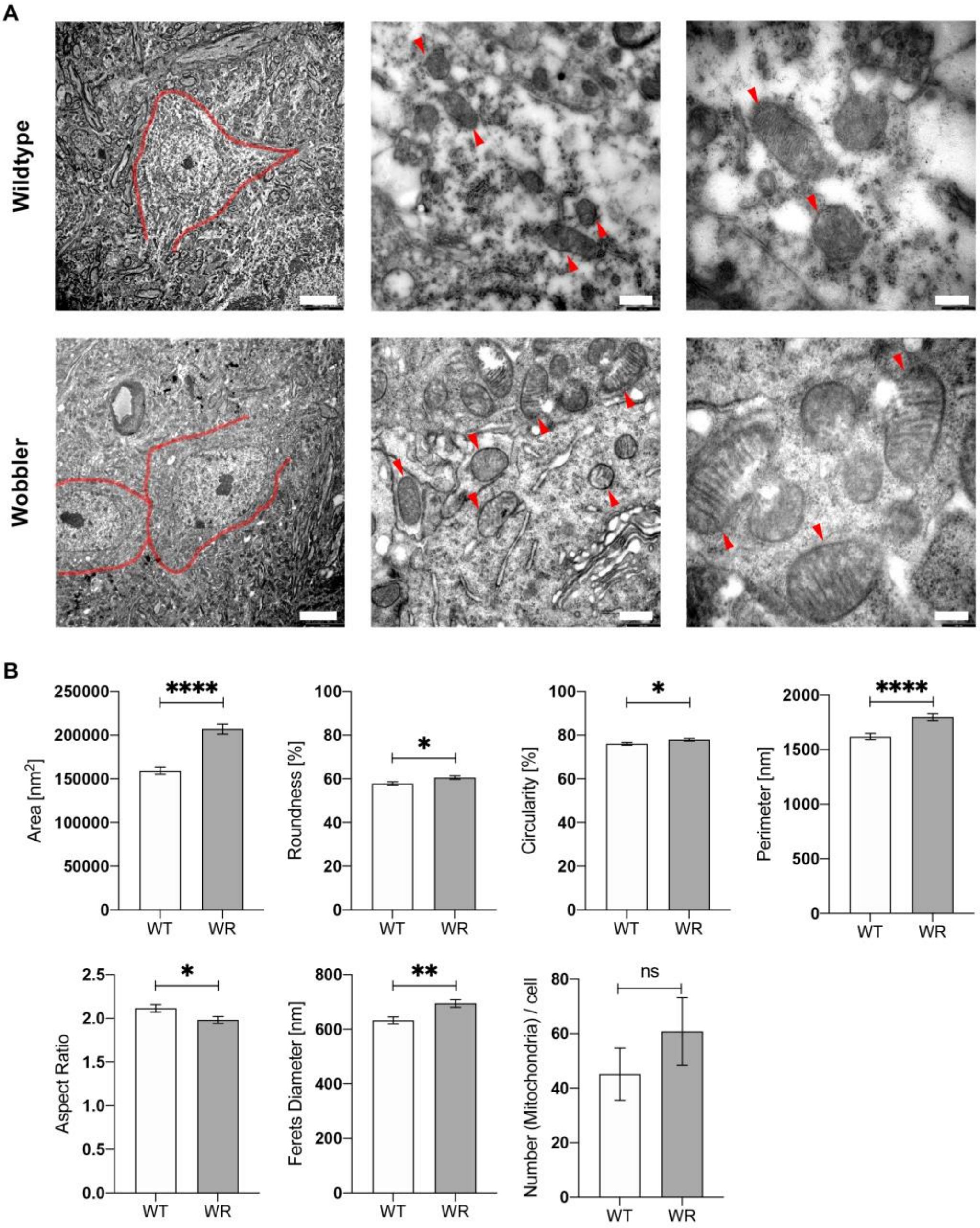

Figure S1. Larger and Rounder Mitochondria of Wobbler Mice Motor Neurons at p20. Transmission electron microscopy of cervical spinal cord of wildtype and wobbler mice at p20. (A) Overview images of motor neurons (red border) and magnified single mitochondria (red arrowheads), indicating an altered mitochondrial morphology in wobbler mice. (B) Measurement of mitochondria with ImageJ revealed a significant increase in area, roundness, circularity, perimeter, and Ferrets diameter combined with a significantly decreased aspect ratio in wobbler mice. Counting mitochondria demonstrated no significant alteration in mitochondrial number per motoneuron. In summary, larger, and rounder mitochondria are present in wobbler motor neurons at p20. Data are presented as means ± SEM. For significance testing, students t-test was performed. Significant differences are indicated by ns>0.05, *p<0.05, **p<0.01, ****p<0.0001. Scale bar = 5µm (left), 500nm (middle), 200nm (right). N=3, n=750 mitochondria per genotype.

Movie S1 (separate file). TEM-tomography of motor neuronal mitochondria of wildtype and wobbler mice. Exemplary video of different planes of reconstructed Z-Stack of a wildtype and a wobbler mitochondrion. Scale bar = 100 nm.

Movie S2 (separate file). Three-dimensional model of motor neuronal wildtype and wobbler mitochondrion. Image alignment and reconstruction by filtered back projection was caried out using the software package IMOD 4.9.7. A 3D model was generated by manual segmentation of the reconstructed image stack with the segmentation feature of 3dMod from the IMOD package. On each image plane individual objects with corresponding contours specific to the mitochondrial structures were assigned and exported as a surface mesh. Small mismatches and failures in the mesh were corrected with the software MeshLab 2020.07 (Institute of Information Science and Technology, Italy) and the final result was rendered by a raytracing algorithm implemented in the software Blender 2.83.2 (Blender Foundation, Netherlands).
